# Treatment-Resistant Schizophrenia: Genetic and Neuroimaging Correlates

**DOI:** 10.3389/fphar.2019.00402

**Published:** 2019-04-16

**Authors:** Antonio Vita, Alessandra Minelli, Stefano Barlati, Giacomo Deste, Edoardo Giacopuzzi, Paolo Valsecchi, Cesare Turrina, Massimo Gennarelli

**Affiliations:** ^1^Department of Mental Health and Addiction Services, ASST Spedali Civili, Brescia, Italy; ^2^Department of Clinical and Experimental Sciences, University of Brescia, Brescia, Italy; ^3^Department of Molecular and Translational Medicine, University of Brescia, Brescia, Italy; ^4^Genetic Unit, IRCCS Istituto Centro San Giovanni di Dio Fatebenefratelli, Brescia, Italy

**Keywords:** treatment resistant schizophrenia (TRS), genetic, pharmacogenetic, pharmacogenomic, neuroimaging, precision medicine

## Abstract

Schizophrenia is a severe neuropsychiatric disorder that affects approximately 0.5–1% of the population. Response to antipsychotic therapy is highly variable, and it is not currently possible to predict those patients who will or will not respond to antipsychotic medication. Furthermore, a high percentage of patients, approximately 30%, are classified as treatment-resistant (treatment-resistant schizophrenia; TRS). TRS is defined as a non-response to at least two trials of antipsychotic medication of adequate dose and duration. These patients are usually treated with clozapine, the only evidence-based pharmacotherapy for TRS. However, clozapine is associated with severe adverse events. For these reasons, there is an increasing interest to identify better targets for drug development of new compounds and to establish better biomarkers for existing medications. The ability of antipsychotics to improve psychotic symptoms is dependent on their antagonist and reverse agonist activities at different neuroreceptors, and some genetic association studies of TRS have focused on different pharmacodynamic factors. Some genetic studies have shown an association between antipsychotic response or TRS and neurodevelopment candidate genes, antipsychotic mechanisms of action (such as dopaminergic, serotonergic, GABAergic, and glutamatergic) or pharmacokinetic factors (i.e., differences in the cytochrome families). Moreover, there is a growing body of literature on the structural and functional neuroimaging research into TRS. Neuroimaging studies can help to uncover the underlying neurobiological reasons for such resistance and identify resistant patients earlier. Studies examining the neuropharmacological mechanisms of antipsychotics, including clozapine, can help to improve our knowledge of their action on the central nervous system, with further implications for the discovery of biomarkers and the development of new treatments. The identification of the underlying mechanisms of TRS is a major challenge for developing personalized medicine in the psychiatric field for schizophrenia treatment. The main goal of precision medicine is to use genetic and brain-imaging information to improve the safety, effectiveness, and health outcomes of patients via more efficiently targeted risk stratification, prevention, and tailored medication and treatment management approaches. The aim of this review is to summarize the state of art of pharmacogenetic, pharmacogenomic and neuroimaging studies in TRS.

## Introduction

Schizophrenia is a disabling disease and many patients who are affected will not be able to achieve their goals in most areas of life. Schizophrenia outcome is quite heterogeneous, with a course of illness characterized by different trajectories (Van Eck et al., 2018). Antipsychotic medication has revolutionized schizophrenia treatment, but approximately one-third of patients show scarce or no response to these drugs (Kane, 2012). The efficacy of antipsychotics for the initial treatment of psychosis is now well established and early antipsychotics introduction in first episode psychosis seems also to improve the long-term course of schizophrenia. Moreover, the effectiveness of antipsychotics maintenance treatment in schizophrenia management and in the relapse prevention represents a therapeutic practice supported by strong data (Goff et al., 2017). The Remission in Schizophrenia Working Group (RSWG) established schizophrenia clinical remission criteria, through the cut-off severity of some characterizing symptoms of the disease (Andreasen et al., 2005). However, clinical remission criteria, while necessary, are not sufficient to explain schizophrenia full functional remission, not taking into account other essential elements of recovery, such as: cognitive performance, depressive symptoms, experiences and daily functioning, quality of life and personal satisfaction (Zipursky and Agid, 2015). Indeed, in the past, more attention was focused on positive symptoms, giving less weight to negative symptoms, cognitive and psychosocial functioning (Vita and Barlati, 2018). It is now well established that positive symptoms explain only a small part of the variance of psychosocial functioning and that the greatest contribution to the functional outcome of schizophrenia is given by negative symptoms, cognitive and social cognitive impairment, as well as anxiety and depression (Galderisi et al., 2014, 2016). Despite the presence of effective antipsychotic drugs and the introduction of evidence-based psychosocial interventions, the course of schizophrenia is characterized by the alternation of remissions and relapses and only a few patients are classified as meeting recovery criteria (Zipursky and Agid, 2015). All this evidence leads to the conclusion that, regardless of the crucial role of antipsychotics, some patients who don’t achieve clinical and functional recovery are defined as treatment-resistant schizophrenia (TRS) patients. Epidemiological data from the scientific literature report that approximately 30% of schizophrenic patients will develop TRS during the course of their disease (Kane et al., 1988; Elkis and Buckley, 2016). The first definitions of TRS were mostly based on the persistence of positive symptoms, despite an adequate antipsychotic treatment for doses and duration (Itil et al., 1966). However, the most commonly used TRS definition in clinical and research fields remains that of Kane’s clozapine study (Kane et al., 1988). However, it has become clear that there is a need to revise Kane’s resistance criteria, or some their variants, giving more attention to psychosocial functioning and not only to positive symptoms. Suzuki et al. (2012) proposed a broad definition of TRS and suggested the criteria include a failure to respond to two adequate doses and durations of antipsychotic treatment. Furthermore, the authors also recommended a comprehensive functional assessment. Recently, the National Institute for Health and Clinical Excellence (NICE) has defined the criteria for the TRS as an insufficient response to at least two different sequential antipsychotic drugs at appropriate doses and taken for an appropriate period of time (Nice Guideline, 2014).

Despite some efforts to standardize the resistance criteria of schizophrenia, there is considerable discrepancy in current clinical approaches. In addition to the NICE criteria, TRS definitions have been proposed by other relevant treatment guidelines, such as: the American Psychiatric Association (APA) (Lehman et al., 2004), the Texas Medication Algorithm Project (Moore et al., 2007), the Schizophrenia Patient Outcome Research Team (PORT) (Buchanan et al., 2010), the World Federation of Societies of Biological Psychiatry Guidelines (Hasan et al., 2012), and the International Psychopharmacology Algorithm Project (IPAP)^1^. All these TRS definitions are different and exposed to a wide range of interpretations, potentially leading to inconsistent clinical management and inaccurate treatment (Howes et al., 2017). Furthermore, even in research field a wide variety of TRS criteria have been applied in different studies. Variation in criteria limits studies comparison, complicates finding interpretation and their replication. Heterogeneity of study designs and populations, including less restrictive definitions of treatment resistance, may contribute to these inconsistencies (Howes et al., 2017). To address this issue, the Treatment Response and Resistance in Psychosis (TRRIP) Working Group has developed consensus criteria and guidelines on TRS, providing a fixed point for research and clinical translation (Howes et al., 2017).

### Aim of the Review

Currently, it is not possible to predict those patients will or will not respond to antipsychotic treatment, and there is a growing interest in identifying new targets for drug development projects and better response biomarkers for current medications. Various levels of evidence have shown that treatment response and resistance in schizophrenia may be associated with certain genetic factors and brain abnormalities (Lally et al., 2016; Mouchlianitis et al., 2016). It is plausible that both neurodevelopmental and neurodegenerative factors may contribute to TRS, in terms of structural, functional brain abnormalities, neurochemical abnormalities or dysregulated gene expression (Elkis and Buckley, 2016). From this perspective, the aim of this review is to summarize the genetic and neuroimaging correlates associated with TRS, to uncover the underlying neurobiological mechanisms of such resistance and to find methods or markers for the early detection of this group of patients. In particular, this review aimed to provide an integrated point of view between genetics and neuroimaging regarding the possible causes of TRS.

## Materials and Methods

### Search Strategy

Electronic searches were performed using MEDLINE/PubMed, PsycINFO, and EMBASE databases combining the following search terms: “schizophrenia,” “pharmacogenetics,” “pharmacogenomic,” “candidate gene study,” “genome wide association study,” “GWAS,” “neuroimaging,” “Positron Emission Tomography – PET,” “Single Photon Emission Computed Tomography – SPECT,” “functional Magnetic Resonance Imaging – fMRI,” “Magnetic Resonance Spectroscopy – MRS,” “typical or first-generation antipsychotics – FGAs,” “atypical or second-generation antipsychotics – SGAs,” “response,” “resistance,” and “refractory.” Detailed combinations of the above search terms are available from the authors on request. Two of the authors (SB, AM) independently reviewed the database to avoid mistakes in the selection of articles. In addition, the reference lists of the included articles were carefully hand-searched to identify other studies of possible interest.

### Selection Criteria

All the studies, meta-analyses, and review articles on pharmacogenetics, pharmacogenomic, structural and functional neuroimaging related to TRS published until June 2018 were included. Studies were included if they met the following criteria: (a) being an original paper published in a peer-reviewed journal, (b) being an English language paper, and (c) involving subjects with TRS, defined according to established international criteria. When the inclusion criteria for TRS were not clearly defined, the study was excluded. Pharmacogenetics or neuroimaging studies on pharmacokinetics or on antipsychotic side effects were not considered.

## Results

### Brain Structural and Functional Abnormalities in Schizophrenia

Since the first MRI study of schizophrenia, the use of this technique allowed the quantification of gray (GM) and white matter (WM) and the measurement of discrete, cortical and subcortical brain structures (Smith et al., 1984). Early morphological studies of schizophrenia primarily assessed specific brain regions of interest (ROIs) (Wible et al., 2001). More recently, functional neuroimaging has provided a direct way of investigating regional brain activity and the pathophysiology of schizophrenia *in vivo*. The presence of multiple small structural brain abnormalities in schizophrenia is now well established (Vita et al., 2015). Results about the progressive brain changes over time in schizophrenia are controversial, and the potential confounding effects of antipsychotics on brain structure is still under discussion. The presence of multiple structural brain abnormalities has been demonstrated by a large number of computed tomography (CT) and MRI studies in the past 40 years and confirmed by several meta-analytic reviews (Olabi et al., 2011; Fusar-Poli et al., 2013; Haijma et al., 2013; Vita et al., 2015). These are predominantly evident in some cerebral regions, such as the ventricular system, cortical GM and subcortical regions (Shenton et al., 2001). Reductions in whole brain measures (3%) and GM volume (2%), primarily in the frontal and temporal lobes, and enlargement of the lateral ventricles (16%) are among the most replicated findings. A small but significant reduction was also found in the WM (1%) (Haijma et al., 2013). A more exhaustive examination of regional brain structural abnormalities has been accomplished by voxel-based morphometry (VBM) studies, which confirmed earlier observed patterns of distributed GM reductions in the bilateral medial frontal and temporal regions, inferior parietal lobe, limbic and striatal regions, insula, thalamus, and basal ganglia (Bora et al., 2011; Palaniyappan et al., 2012). In their VBM meta-analysis, Bora et al. (2011) indicated a reduction in GM density in the dorsal and rostral anterior cingulate cortex (ACC), left lateral prefrontal areas, superior frontal gyrus, and orbitofrontal and fusiform regions.

Additionally, studies of WM tracts showed evidence of disorganization and an absence of alignment in white fiber bundles in frontal and temporoparietal brain regions and a reduction in WM diffusion anisotropy in schizophrenia subjects (Burns et al., 2003; Davis et al., 2003). More recently, diffusion tensor imaging (DTI) studies have identified several regions with decreased fractional anisotropy, reflecting altered WM connections and supporting the “disconnection model of schizophrenia” (Ellison-Wright and Bullmore, 2009; Crossley et al., 2017).

Regarding functional neuroimaging, this technique has been used to study patterns of increased or decreased activity within the brains of subjects with and without schizophrenia during rest and various assigned behavioral tasks; these studies have revealed that the affected parts of the central nervous system (CNS) are not contained within a single brain region but rather lie within neural networks that include numerous brain regions (Gur and Gur, 2010). Functional brain abnormalities in schizophrenia include alterations in information storage and retrieval by the dorsolateral prefrontal cortex (dlPFC), alterations in inhibitory responses to sensory stimuli by the ACC, deficits in memory encoding and retrieval by the hippocampus, alterations in sensory information reception and integration by the thalamic nuclei, primary sensory cortices and multimodal cortices and impairments in performance of cognitive tasks associated with the basal ganglia, thalamus, and cerebellum (Wright et al., 2000; Davis et al., 2003; Glahn et al., 2005). fMRI studies showed patterns of widespread alterations in task-induced activity, which overlap with patterns of GM findings leading to one consistent results that is a decreased activation of frontal regions during cognitive tasks (Glahn et al., 2005; Minzenberg et al., 2009). However, this finding surprisingly was not consistently replicated when SPECT semiquantitative assessments were replaced by fMRI (Callicott et al., 2003). Furthermore, functional studies of social cognition and emotional processing suggested altered responses of the amygdala and hippocampus, potentially with respect to aversive stimuli (Li et al., 2010). The pathogenesis of structural and functional alterations in schizophrenia is still poorly understood, and only an ongoing integration of structural data with functional imaging may offer more insight in this field (Gur and Gur, 2010). Several longitudinal and cross-sectional MRI studies examined the meaning of such brain abnormalities, their static or progressive nature and their time of occurrence (van Haren et al., 2008). Finally, some recent studies have been reported that brain changes appear to be especially relevant in the first years of illness (Schnack et al., 2016; van Haren et al., 2016), although other studies have not confirmed these findings (Roiz-Santiañez et al., 2015).

### Brain Abnormalities in Schizophrenia: Are They Reversible or Not?

In the last two decades, several studies have been conducted for understanding if reported abnormalities could reversible or not with some interventions. Among the first investigations, Keshavan and collaborators have shown an ameriolation of GM volume deficits in the superior temporal cortex and hippocampus in schizophrenia patients (Keshavan et al., 1998). In a more recent longitudinal study in a subgroup of first-episode psychosis patients who presented a remitting course after approximately 18 months, has been obtained a reversal of temporal lobe GM deficits (Schaufelberger et al., 2011). These results are consistent with other findings about the brain volume deficits reversibility in association with schizophrenia symptom improvement (de Castro-Manglano et al., 2011; Roiz-Santiáñez et al., 2015; Torres et al., 2016).

The longitudinal MRI studies further suggest that the degree of progression of brain structural abnormalities over the course of schizophrenia partially occurs with of the chronic antipsychotic usage. However, according to Vita et al. (2015) the class of antipsychotic is a key variable, because of more progressive GM loss correlates with higher mean daily antipsychotic intake in patients treated with at least one FGA, whereas less progressive GM loss correlates with higher mean daily antipsychotic intake in patients treated with SGAs only.

In addition, several neuroimaging studies on non-pharmacological intervertions in schizophrenia, indicated that cognitive remediation improves brain activation in two main areas: the prefrontal and thalamic regions. Accordingly, it has been suggested a positive effect of cognitive remediation on brain functioning in terms of the functional reorganization of neural networks, and structural changes were described both in GM and WM, confirming a neuroprotective effect of cognitive remediation (Penadés et al., 2017, 2019). Promising results have been also obtained with cognitive behavioral therapy (Mason et al., 2016, 2017) and physical aerobic exercise (Svatkova et al., 2015; Malchow et al., 2016).

On the other hand, several well-conducted MRI investigations have provided evidence that structural brain abnormalities associated with the diagnosis of schizophrenia may progress from the first psychotic episode to chronic disease stages, particularly during the initial few years after illness onset, even if these irreversible brain changes are restricted to subgroups of patients with an unremitting disease course and poorer outcome (Andreasen et al., 2013; Cannon et al., 2015).

Overall, literature data are controversial and further studies will be needed to better understand if brain abnormalities are reversible and which are not, at which stage of illness and with which type of intervention. Despite these limitations, most robust results demonstrate a reversibility of some brain abnormalities, particularly in the early stages of the illness, in relation to schizophrenia outcome.

### Brain Structural Abnormalities in Treatment-Resistant Schizophrenia

Ventricular enlargement is one of the variables most studied in TRS. Early CT studies showed an inverse relationship between degree of ventricular enlargement and antipsychotics treatment response (Weinberger et al., 1979; Friedman et al., 1992; Mitelman and Buchsbaum, 2007). These findings were confirmed by subsequent CT studies, using also morphometric techniques, such as ventricular brain ratio (VBR). Over the last three decades, CT and then MRI cross sectional studies including chronic patients have found an association between ventricular enlargement and poor outcome (Friedman et al., 1992; Mitelman and Buchsbaum, 2007). In particular, studies in patients whose illness is progressive and resistant to treatment have shown abnormalities such as ventricular enlargement and decrease in GM (Mitelman and Buchsbaum, 2007; Mitelman et al., 2010). Many subsequent studies tried to replicate these findings, but a first meta-analysis of these early studies as well as a critical review of this subject found no relationship between ventricular enlargement and treatment response in schizophrenia patients (Borgio et al., 2010). Several longitudinal studies conducted on chronic patients (Davis et al., 1998) or first psychotic episode patients (Mitelman and Buchsbaum, 2007) confirmed these structural changes in the brain and found that they were progressive over the course of illness. In particular, in the first study just mentioned, it has been shown that “Kraepelinian patients” manifested left-sided ventricular enlargement compared to treatment responsive patients followed over the same 5-year follow-up period (Davis et al., 1998).

In an early ROI MRI study, Lawrie et al. (1995) found that poorly responsive patients had lower volumes of most brain structures than treatment responders, but no brain-imaging variables were statistically related to the outcome. In a later MRI study performed by the same research group, TRS patients showed a tendency to greater atrophy than those were treatment responsive (Lawrie et al., 1997). In this study, patients were selected as dichotomous groups (matched for age, sex, and illness duration) of treatment-responsive and TRS patients using a descriptive criteria: responsive patients showing a marked reduction of symptoms and being able to return to the same social situation; resistant cases showing severe residual symptoms and requiring long-term institutional care.

In addition, in the MRI study performed by Buchsbaum et al. (2003), schizophrenia patients with a good outcome had larger relative mean putamen size, most marked for the dorsal putamen and right hemisphere, than poor outcome patients or normal controls. The authors suggested that the expansion of putamen size may be a physiological correlate of antipsychotic responsiveness and that small putamen size at disease onset may be a predictor of poor outcome (Buchsbaum et al., 2003).

The GM decrease in total volume or localized reductions in certain regions, such as the frontal, temporal and occipital cortexes and ventral thalamus were identified in very poor outcome schizophrenia patients (Mitelman and Buchsbaum, 2007). Overall, TRS showed a GM reduction particularly in frontal, temporal, and occipital regions (Molina et al., 2008; Quarantelli et al., 2014; Ahmed et al., 2015; Anderson et al., 2015) compared with healthy subjects and a GM reduction particularly in frontal regions (Lawrie et al., 1995; Mitelman et al., 2005; Zugman et al., 2013; Quarantelli et al., 2014; Anderson et al., 2015) compared with responders.

Recent studies using the VBM technique found significant differences between TRS patients and non-TRS patients. Zugman et al. (2013) showed that TRS patients showed a decrease in cortical thickness in all brain regions in comparison to healthy controls, with a marked decrease in dlPFC thickness when compared to responder patients. Quarantelli et al. (2014) showed more pronounced degrees of GM atrophy in TRS patients, both compared to healthy controls and to responders schizophrenic patients. Moreover, in an MRI cross-sectional study, Anderson et al. (2015) found GM reductions both in TRS and in clozapine-resistant schizophrenia (“ultra-TRS”) patients (Anderson et al., 2015). In a longitudinal MRI study of TRS patients switched to clozapine, Ahmed and colleagues found a progressive regional brain volume loss in the prefrontal cortex (PFC) and in the periventricular area and a global cortical thinning, compared with healthy controls (Ahmed et al., 2015). However, due to the heterogeneity of these studies, two recent systematic reviews showed contrasting results concerning reductions in GM in TRS patients (Nakajima et al., 2015; Mouchlianitis et al., 2016).

Abnormalities of WM have been reported in the frontal, parietal and temporal regions and have been associated with poor outcomes (Mitelman and Buchsbaum, 2007; Molina et al., 2008). Moreover, in a DTI study, TRS patients showed an enlargement of the posterior corpus callosum, particularly the splenium, and widespread disruptions to WM tract integrity compared with healthy subjects (Holleran et al., 2014) and enlarged WM volumes compared with treatment-responsive patients (Molina et al., 2008; Anderson et al., 2015). In addition, connectivity in TRS patients, compared to non-TRS patients, showed a reduction in ventral striatum and substantia nigra connections, and an alteration in the distribution of corticostriatal connections (White et al., 2016). A recent systematic review (Mouchlianitis et al., 2016) showed an increase in basal ganglia WM in TRS, compared to schizophrenia patients who were responsive to treatment.

In summary, TRS patients show greater GM reduction, especially in frontal regions, and an increase in WM volume. Despite these findings have been replicated, more research is need to identify a neuroimaging profile able to recognize subject with higher risk to not respond to antipsychotics and consequently with higher vulnerability to develop TRS.

### Brain Functional Abnormalities in Treatment-Resistant Schizophrenia

Functional neuroimaging techniques offer indirect ways of investigating brain activity *in vivo*. Functional neuroimaging data showed that a lower striatal metabolism before antipsychotics treatment was a predictor of a good clinical response and that responders patients showed a greater increase in striatal metabolism after antipsychotics therapy (Buchsbaum et al., 1992a,b; Bartlett et al., 1998). A recent extensive review (Nakajima et al., 2015) pointed out a pattern of hypometabolism in the PFC and hypermetabolism in the basal ganglia. Similar results support these findings; for example another recent systematic review (Mouchlianitis et al., 2016) showed decreased metabolism in frontotemporal regions and increased perfusion in the basal ganglia in TRS. Moreover, some research groups investigated whether disruptions in resting-state functional connectivity were associated with TRS (Paul and Sharfman, 2016; McNabb et al., 2018). Recently, in a fMRI study, Ganella et al. (2017) assessed functional brain networks abnormalities in TRS patients in comparison with healthy subjects, showing a global brain functional connectivity reduction in patients. In particular, this study revealed a decrease in temporal, occipital, and frontal region (Ganella et al., 2017). Other studies focusing con brain connectivity performed with different paradigms lead to similar conclusion of a general functional connectivity decrease (Wang et al., 2015; White et al., 2016; Vanes et al., 2018).

### Neurotransmission in Treatment-Resistant Schizophrenia: Findings From Molecular Neuroimaging Studies

Molecular neuroimaging provides a direct way of investigating brain activity. In addition, various levels of evidence have shown that treatment response and resistance in schizophrenia can be associated genetic factors influencing gene involved in the pharmacokinetics and pharmacodynamics of anti-psychotic drugs. Indeed, a single nucleotide polymorphism (SNP), can introduce a missense substitution, thus altering the encoded protein and its function, or can affect non-coding regulatory regions (promoter, 3′UTR, intronic regions), influencing RNA transcription and splicing. Additionally, alterations in the number of gene copies (CNV) can result in increased or decreased levels of active protein present in the cells. Thus, the combination of SNPs and/or CNVs in each individual determines a unique profile for the activity of genes with an impact on the response to different drugs.

To understand the possible causes of brain changes in TRS, genetics and neuroimaging, i.e., “imaging genetics,” provides an integrated point of view. These studies suggest that TRS is related to a variety of alterations and pathophysiological mechanisms that implicate different neurotransmitter systems. In particular, dopaminergic, serotonergic, glutamatergic, and GABAergic dysregulation, as well as numerous other alterations affecting other neural systems, have been demonstrated to play a relevant role in treatment resistance.

#### Dopaminergic System

The dopaminergic system has been studied for a long time in schizophrenia, since the dopamine hypothesis was formulated in the 1960s after the discovery of the antipsychotic actions of chlorpromazine, and it was enormously useful as a heuristic principle for the interpretation of the phenomenology features of schizophrenia. The dopamine hypothesis assumes that hyperactivity of dopamine D2 receptor neurotransmission in subcortical and limbic brain regions contributes to the positive symptoms, while negative and cognitive symptoms can be attributed to hypofunctionality of dopamine D1 receptor neurotransmission in the prefrontal cortex (Nakata et al., 2017). Indeed, antipsychotic (D2 antagonistic) treatment reduces positive psychotic symptoms in most patients but there is considerable heterogeneity in treatment response with roughly one-third of patients showing insufficient clinical response (Lindenmayer, 2000). Furthermore, there is variability regarding the time to clinical response after antipsychotic treatment onset (Emsley et al., 2006) and variability regarding the re-emergence of symptoms despite sufficient D2-receptor blockade (Rubio and Kane, 2017). In this context, dopamine receptors are among the main targets of antipsychotic drugs and TRS patients have shown reduced striatal dopamine synthesis capacity compared who had responded to antipsychotic treatment (Demjaha et al., 2012). The same research group found that patients with high levels of glutamate in the ACC (as measured by MRS) and with normal presynaptic dopamine synthesis (as measured by PET) showed a poor antipsychotic treatment response (Demjaha et al., 2014). Taken together, these results allow to hypothesize a “non-dopaminergic” subtype of schizophrenia (Howes and Kapur, 2014). As compared to the “hyperdopaminergic” subtype, characterized by prominent striatal dopamine synthesis and release capacity, the “non-dopaminergic” subtype exhibited normal dopaminergic function, and the disorder symptoms were not related to dopaminergic transmission. This classification based on a neurobiological mechanism shows several advantages: it could lead to the identification of PET scanning tests that guide treatment choice at illness onset and could provide a basis for research in order to develop new treatment options (Howes and Kapur, 2014). Some studies suggested that glutamatergic alterations may underlie the “non-dopaminergic subtype” of schizophrenia. More specifically, treatment responders seem to have more marked dopaminergic aberrations, whereas treatment non-responders seem to have more marked glutamatergic abnormalities (Howes et al., 2015).

Moreover, Roberts et al. (2009) examined dopaminergic synapses at the electron microscopic level in postmortem caudate of non-TRS and TRS patients. Despite the results of this study should be confirmed by replication, because of the small sample size, a good treatment response has been correlated with higher density of dopaminergic synapses, which supports a biological basis for TRS (Roberts et al., 2009).

Given the central role of the dopaminergic neurotransmitter system in the antipsychotic response, related genes have been widely investigated in studies on treatment response/resistance in schizophrenia, focusing in particular on gene variations encoding the dopamine D2 (*DRD2*) and D3 (*DRD3*) receptors (Arranz et al., 2011; Reynolds, 2012a; Brandl et al., 2014). Among the single SNPs in the *DRD2* gene, the most investigated is rs1800497 (Taq1A). The A1 allele of the TaqIA polymorphism, has been shown to reduce gene expression and therefore has also been hypothesized to influence treatment response (Brandl et al., 2014). However, studies performed to date have reported inconsistent findings (Schäfer et al., 2001; Lencz et al., 2006; Kohlrausch et al., 2008). In addition, subsequent studies demonstrated that TaqIA is located in exon 8 of ankyrin repeat and kinase domain containing 1 (*ANKK1*) gene, located close to *DRD2*, where it causes a non-conservative amino acid substitution (Neville et al., 2004; Lucht and Rosskopf, 2008). It is not clear if ANKK1 gene plays any role in neuropsychiatric disorders and drug response variability previously associated with Taq1A or if this polymorphism is in linkage disequilibrium (LD) with some other variants in DRD2 gene actually responsible for the effects on the dopamine transporter. For other *DRD2* polymorphisms, such as TaqIB, Ser311Cys, or A-241G, few studies have described associations (Lane et al., 2004; Hwang et al., 2005; Lencz et al., 2006; Zhang et al., 2010), but contrasting data and an absence of replication make further investigations necessary.

In the *DRD3*, the Gly9 variant of the Ser9Gly polymorphism changes D3 receptor density (Jeanneteau et al., 2006; Prieto, 2017). Consequently, the impact of this variant concerning antipsychotics response has been widely investigated (Arranz et al., 2011; Reynolds, 2012a; Brandl et al., 2014), but as with other receptor genes, such as *DRD1, DRD4, DRD5*, inconclusive findings have been obtained (Hwang et al., 2010; Brandl et al., 2014; Lally et al., 2016).

With a more complex approach, Pergola et al. (2017) studied genetic variants in relation to the *DRD2* gene co-expression pathway in association with working memory behavior, the related brain activity and the response to treatment. This study showed that a *DRD2* co-expression gene set enriched for protein-coding genes associated with schizophrenia modulates PFC function during working memory and response to D2 antagonist antipsychotics. These data revealed important findings; *DRD2* co-expression can parse schizophrenia risk genes into biological pathways associated with intermediate phenotypes as well as with clinically meaningful information.

Another interesting and well-characterized *DRD2* polymorphism is the rs1076560 since it was associated with gene function and response to treatment. This SNP is a regulatory variant that decreases the expression ratio of *DRD2* short isoform relative to the long isoform (Zhang et al., 2007). Moreover, it has also been associated with response to antipsychotic treatment, both alone and in interaction with another functional polymorphism rs1130233 within the serine/threonine kinase 1 (*AKT1*) gene pertaining to a cAMP independent D2 signaling pathway (Blasi et al., 2011). Furthermore, it has also been associated with several schizophrenia-related phenotypes in healthy individuals, such as increased activity of striatum and prefrontal cortex and reduced performance in working memory and attentional control tasks (Blasi et al., 2011; Colizzi et al., 2015).

In conclusion, pharmacogenetic studies carried out on genes involved in the dopaminergic system to date, have moderate sample sizes and examined single or few polymorphisms in selected candidate genes. Overall, most results remain conflicting, and most associations fail to be replicated in large Genome Wide Association Studies (GWAS) and meta-analyses (Liou et al., 2012; Gressier et al., 2016; Hettige et al., 2016; Terzić et al., 2016; Koga et al., 2017). The reported effect sizes for genetic variants associated with antipsychotics are modest, and none of them effectively predict the treatment response (Pouget et al., 2014). However, such modest effect sizes are not surprising, given the complexity and polygenicity of this endophenotype. Rare variants in dopamine-related genes also seem to influence the response to antipsychotics, as suggested by a recent analysis of whole exome sequencing data in a large cohort of TRS patients (Ruderfer et al., 2016).

#### Serotonergic System

A plethora of serotonin receptors, as well as transporter gene polymorphisms, have been suggested as being involved in the mechanism of action of antipsychotic responses in schizophrenia. Although several studies have reported significant associations, these results have not been consistently replicated. Of the 5-HT receptors, the 5-HT2A receptor has been the most studied in schizophrenia and relative treatments. The greatest number of studied were focused on two polymorphisms, that are the 102T/C (rs6313), a synonymous coding region SNP, and 1438A/G (rs6311), a promoter SNP that is in complete linkage with 102T/C and reportedly has functional effects on gene expression. Moreover, several studies have investigated a further functional non-synonymous coding region SNP, 452His/Tyr (rs6314). This SNP has been found associated with response to antipsychotic treatments, either alone (Arranz et al., 2011) or in combination with the *DRD2* polymorphism rs1076560 described above in the dopaminergic system section (Blasi et al., 2015). A better response to antipsychotics is reported for schizophrenia patients with the combination of rs1076560 T and rs6314 CC genotypes, in two small cohorts. This results suggest that the effect of 5-HT2A variants on treatment response could be influenced by a complex interaction with D2 receptor variants, given that both receptors share the same intraneuronal molecular pathway (de Bartolomeis et al., 2013).

In general, contrasting results have emerged; consequently, there are no clear findings regarding the pharmacogenetics of antipsychotics and 5-HT2A receptors. In detail, functional variants of the serotonin 5-HT2A receptor gene were associated with less amelioration in psychotic symptoms following the treatment with clozapine (Arranz et al., 2011), olanzapine (Ellingrod et al., 2002) and risperidone (Lane et al., 2002), but negative associations were also reported for the same drugs (Masellis et al., 1995; Malhotra et al., 1996a; Lin et al., 1999; Thomas et al., 2008).

In early studies, the 5-HT2C receptor has also been shown to have some associations of potentially functional SNPs with antipsychotic response with inconclusive findings (Masellis et al., 1995; Sodhi et al., 1995; Malhotra et al., 1996b; Rietschel et al., 1997; Ellingrod et al., 2002; Thomas et al., 2008; Liu et al., 2010). However, most part of pharmacogenetic studies of 5-HT2C gene have reported positive associations with the metabolic side effects. Concerning the remaining 5-HT receptor genes, sparse data and unsettled conclusions are available in relation to the clinical consequences of antipsychotic treatment (Yu et al., 1999; Masellis et al., 2001; Houston et al., 2007; Gu et al., 2008; Wei et al., 2009; Takekita et al., 2016).

The great majority of pharmacogenetics studies performed to date in psychiatric field, investigated the neuronal 5-HT transporter gene (HTT; *SLC6A4*). This mutation has a functional ins/del promoter polymorphism (HTTLPR) in which the short allele (del) leads to a reduction of transporter activity of the HTT protein due to lower expression. However, during the last decade, it has been critically noted that the analysis of 5-HTTLPR is incomplete because other polymorphisms have been identified in the proximity of the Ins/Del locus, such as rs25531, rs25532, rs2020933, and a 17-bp variable tandem repeat in the second intron (STin2) (Bonvicini et al., 2010). *SLC6A4* polymorphisms have been extensively examined in mood disorders and antidepressant treatment, while little work has been performed in relation to antipsychotic response, with few significant results (Arranz et al., 2000; Bozina et al., 2007; Wang et al., 2007; Dolzan et al., 2008; Kohlrausch et al., 2010).

Although there are inconsistent results concerning TRS/response to antipsychotics and serotonin system polymorphisms, there are some exciting data coming from neuroimaging genetic studies that have confirmed the crucial role of serotonergic signaling in the antipsychotic treatment response. A study by Blasi et al. (2013) showed that rs6314 of the 5-HT2A gene affects 5-HT2AR expression and functionally contributes to the genetic modulation of endophenotypes of schizophrenia, such as higher-level cognitive behaviors and related prefrontal activity, as well as to olanzapine response. In particular, this functional brain imaging study (Blasi et al., 2013) indicated that individuals carrying the T allele have overstated prefrontal responses during working memory and attentional control tasks and also impaired cognitive behavioral performance. Moreover, schizophrenia patients who carry the T allele, compared to those who do not, have an attenuated improvement in negative symptom scores after 8 weeks of olanzapine treatment.

#### Glutamatergic/GABA Systems

The contribution of glutamatergic/GABA systems to the development of schizophrenia has been hypothesized for many years. To date there has been a growing body of evidence showing alterations in glutamatergic neurotransmission in relation to several aspects of the disorders. This evidence led to several studies investigating the role of these systems in antipsychotic treatment outcomes.

In a proton MRS (1H-MRS) study in first-episode psychosis, Egerton et al. (2012) found elevated glutamate levels in the ACC in patients who had persistent psychotic symptoms despite antipsychotic treatment, relative to responders (Egerton et al., 2012). In the same year, in a (18F-DOPA) PET study, Demjaha et al. (2012) showed that TRS patients were characterized by elevated ACC glutamate levels. In a later 1H-MRS study they also found that patients with high levels of glutamate in the ACC (as measured by MRS) and with normal presynaptic dopamine synthesis (as measured by PET) showed a poor antipsychotic treatment response (Demjaha et al., 2014). In authors opinion, these data suggest that treatment resistance in schizophrenia is associated with a combination of relatively normal striatal dopamine synthesis and elevated ACC glutamate levels (Demjaha et al., 2014).

Taken together, these studies suggest that neuroimaging measures of dopamine and glutamate function might provide a means of stratifying patients with psychosis according to their response to treatment. Therefore, it could be argued that in some patients with schizophrenia, antipsychotic treatment may be ineffective because they do not exhibit the elevation in dopamine synthesis capacity that is classically associated with the disorder.

A recent review summarized that TRS compared to responder patients have more regions with decreased GM and show glutamatergic but no dopaminergic abnormalities (Gillespie et al., 2017). A more recent systematic review has taken in consideration all longitudinal proton MRS studies investigating antipsychotic treatment effect on brain glutamate levels in schizophrenia patients (Egerton et al., 2017). The main finding reported from the authors is that most part of studies described a significant decrease in glutamate metabolites after antipsychotic treatment in at least one brain region. Because of schizophrenia is related with an increase in glutamate metabolites, this data provides some indications that antipsychotics can reduce glutamatergic levels. However, to date the results have shown that this effect are quite small and/or limited to subgroups of patients (Egerton et al., 2017).

Glutamatergic neurotransmission takes place through metabotropic and ionotropic glutamate receptors. The metabotropic receptor (mGluR) family is subdivided into 3 groups, with a total of eight identified subtypes, and the ionotropic receptor family is made of α-amino-3-hydroxy-5-methyl-4-isoxazolepropionic acid (AMPA), *N*-methyl-D-aspartate (NMDA) and kainate receptors (Nakanishi, 1992). While ionotropic receptors mediate fast excitatory transmission at the glutamatergic synapse, ligand binding at metabotropic receptors leads to conformational changes directly or indirectly influencing neurotransmission by second messenger pathways (Kew and Kemp, 2005).

On this basis, glutamate-related genes have been investigated in relation to antipsychotic treatment in schizophrenia. The glutamate metabotropic receptor 3 (*GRM3*) gene has been widely investigated since it modulates signaling through NMDA receptors which are a relevant contributor to the cognitive and negative symptoms of schizophrenia (Maj et al., 2016). Several studies have found an association between *GRM3* and antipsychotic response or treatment resistance (Bishop et al., 2005, 2011, 2015; Fijal et al., 2009; Kaur et al., 2014). The GRM3 gene was found associated to schizophrenia in large GWAS analysis (Schizophrenia Working Group of the Psychiatric Genomics Consortium, 2014) and it encodes for the mGluR3 receptor, with a prominent role in the glutamate signaling in the brain (Cartmell and Schoepp, 2000). Two SNPs in *GRM3* (rs1989796 and rs1476455) resulted associated to TRS in a cohort made mainly of Caucasian individuals with the rs1476455_CC and rs1989796_CC genotypes associated to higher BPRS scores (Bishop et al., 2011). Polymorphisms in this gene were also found associated to worsening after antipsychotic treatment (rs1468412) and improvement in negative symptoms (rs6465084) in first-episode schizophrenia patients (Bishop et al., 2015). Moreover, the SNP rs1468412 showed a synergistic effect with the SNP rs165854 within phosphatidylinositol 4-Kinase Alpha (*PI4KA*) gene influencing antipsychotic response in low-severity schizophrenia patients of Indian origin (Kaur et al., 2014).

Two recent studies have supported the glutamate system as a potential mechanism of the response to risperidone, showing interesting evidence concerning the glutamate metabotropic receptor 7 (*GRM7*) gene (Stevenson et al., 2016; Sacchetti et al., 2017). In particular, Stevenson et al. (2016) identified an association between two SNPs in *GRM7* (rs2069062 and rs2014195) and an antipsychotic treatment response by a candidate gene analysis in a sample of first episode psychosis patients. In contrast, our group (Sacchetti et al., 2017) has shown a relevant role of rs2133450 as a predictor of an early (2 weeks) response to risperidone in a sample of schizophrenia patients through an original GWAS and a confirmatory analysis carried out on the Clinical Antipsychotic Trials of Intervention Effectiveness (CATIE) schizophrenia study sample (Stroup et al., 2003).

Spurious and contrasting results are available for other genes in the glutamatergic system, such as the glutamate ionotropic receptor delta type subunit 2 (*GRID2*) (Stevenson et al., 2016) and the glutamate ionotropic *N*-methyl-D-aspartate receptor 2B subunit (Hong et al., 2001; Taylor et al., 2016).

Interestingly, a recent analysis of whole exome sequencing data revealed an enrichment for singleton disruptive mutations in 347 gene targets of antipsychotics in a large cohort of TRS patients (Ruderfer et al., 2016). These genes also included genes of the GABAergic/glutamatergic system, such as gamma-aminobutyric acid (GABA) A receptor alpha 5 (*GABRA5*), gamma-aminobutyric acid receptor subunit beta 2 (*GABRB2*), and glutamate ionotropic receptor NMDA type subunit 2A (*GRIN2A*). Finally, NMDA receptor-mediated signaling genes, such as D-amino acid oxidase (*DAO*), protein phosphatase 3 catalytic subunit gamma isoform (*PPP3CC*), and dystrobrevin-binding protein 1 (*DTNBP1*) genes, were associated with both the pathogenic mechanisms of and antipsychotic treatment response in schizophrenia (Reynolds, 2012b; Sacchetti et al., 2013).

The future development of drugs capable of supporting the glutamatergic functions would be of great interest (Carlsson et al., 1999). Based on the *N*-Methyl-D-aspartate receptor (NMDAR) hypofunction hypothesis of schizophrenia (Coyle, 2006; Moghaddam and Javitt, 2012), the setting of pharmacological agents that enhance NMDAR function could provide therapeutic benefits in patients with schizophrenia. Unfortunately, direct activation of NMDARs using traditional orthosteric agonists induces adverse effects such as excitotoxicity and seizures (Puddifoot et al., 2012). Furthermore, treatments with NMDAR obligate co-agonists such as glycine or serine failed to have consistent efficacy across multiple clinical trials (Iwata et al., 2015). More recently, selective NMDAR positive allosteric modulators (PAMs) that enhance receptor function in the presence of the endogenous agonists but are devoid of intrinsic activity have been reported (Hackos et al., 2016). It is possible that NMDAR PAMs could avoid the adverse effects associated with direct activation of NMDARs. The recent development of NMDAR PAMs such as GNE-6901 and GNE-8324 provide proof-of-principle for the development of allosteric modulators of NMDARs, however their poor pharmacokinetic properties and low CNS exposures hinder their uses for *in vivo* studies (Hackos et al., 2016).

In addition to NMDARs all three groups of mGlu receptors have been pursued as putative targets for novel antipsychotics due to their ability to directly alter NMDAR function or other aspects of glutamatergic signaling. The metabotropic glutamate receptors represent a large group of promising targets for novel therapeutics to treat all three symptom domains of schizophrenia (positive, negative, and cognitive symptoms). While many discovery efforts are still in preclinical phases of development, they have yielded several subtype-selective tool compounds with minimal adverse effect profiles and promising preclinical efficacy.

In conclusion, on one hand several data evidenced that glutamatergic drugs are effective for the treatment of schizophrenia, however on the other hand conclusions are somewhat mixed and, where supported by meta-analyses, the effect size is unfortunately modest.

#### Other Systems

Only a few studies have investigated candidate genes not belonging to the major neurotransmission systems and the relationship to antipsychotic responses. Some association studies have focused on genes involved in the transport of various drugs through the blood-brain barrier and multi-drug resistance (e.g., the ATP-binding cassette, ABC, transporter proteins). In particular, *ABCB1, ABCC1* and *ABCB11* were significantly associated with the efficacy of or response to different antipsychotic drugs, including clozapine (Gonzalez-Covarrubias et al., 2016; Mi et al., 2016; Piatkov et al., 2017). Going beyond the candidate gene approach, recent studies have used GWA approaches for hypothesis-free investigation of common genetic factors.

Several GWA studies on TRS failed to identify significant associations (Hettige et al., 2016; Martin and Mowry, 2016; Koga et al., 2017; Wimberley et al., 2017), probably due to small sample sizes. Indeed, when GWA studies were performed in larger cohorts, suggestive associations emerged for various genomic loci involving genes related to immune responses (Liou et al., 2012) or genes involved in neuronal transmission and neurodevelopment (Yu et al., 2018).

Another investigative approach is the use of polygenic risk scores (PRSs) that summarize genome-wide genotype data into a single variable that measures genetic vulnerability to a disorder or a specific trait. Currently, the PRS is also frequently used to follow up a GWAS, testing the prediction of a drug response. To date, no significant results have emerged for TRS (Hettige et al., 2016; Martin and Mowry, 2016; Wimberley et al., 2017). Based on these findings, the use of the PRS for schizophrenia to classify individuals with TRS to date is scarce to be of clinical utility.

Finally, recent advances in sequencing technologies have opened the way for GWA studies and TRS for rare variants. A large sequencing analysis on coding regions (exome) in TRS patients found an excess of disruptive mutations in 347 genes involved in antipsychotic mechanisms of action (Ruderfer et al., 2016). Interestingly, some of these genes, such as calcium voltage-gated channel subunit alpha1 C (*CACNA1C*), glutamate ionotropic receptor NMDA type subunit 2A (*GRIN2A*), AKT serine/threonine kinase 3 (*AKT3*), hyperpolarization activated cyclic nucleotide gated potassium channel 1 (*HCN1*), solute carrier family 1 member 1 (*SLC1A1*) were previously associated with schizophrenia pathogenesis or a specific antipsychotic response (Ryu et al., 2011; Liu et al., 2015; Pers et al., 2016; Kabir et al., 2017; Yu et al., 2018).

Finally, in addition to common genetic variants, rare variants indexed by deletion and duplication burden genomewide, can increase the understanding and clinical management of TRS patients; however, to date, little data are available (Martin and Mowry, 2016).

Table 1 summarizes the literature main findings about structural, functional, molecular and neurochemistry brain abnormalities in TRS.

**Table 1:** Brain abnormalities in TRS: literature main findings.

**Structural abnormalities**
Greater GM reduction, especially in frontal regions, compared to responders
Decrease in dlPFC thickness compared to responders
Greater GM reduction, particularly in frontal, temporal and occipital regions, compared to HC
Decrease in cortical thickness in all brain regions, compared to HC
Widespread increase in WM volume (frontal, parietal, occipital), compared to HC
Increase in basal ganglia WM volume, compared to responders
Enlargement in posterior sections of the corpus callosum (splenium), compared to HC
Widespread disruption in WM tract integrity, particularly in the corpus callosum, compared to HC
**Functional abnormalities**
Decreased metabolism and perfusion in frontal areas, compared to HC
Increased perfusion in the basal ganglia, compared to HC
Global brain functional connectivity reduction, particularly in frontal, temporal and occipital regions, compared to HC
**Molecular and neurochemistry abnormalities**
Reduced striatal dopamine synthesis compared to non-TRS, but no difference from HC
Elevated glutamate concentration in ACC, compared to responders
Increased glutamate and glutamine concentrations in the putamen and decreased in the dlPFC in TRS clozapine responders, compared to first-line antipsychotic responders

*ACC, anterior cingulate cortex; dlPFC, dorsolateral prefrontal cortex; GM, gray matter; HC, healthy controls; TRS, treatment-resistant schizophrenia; WM, white matter.*

### Clozapine in Treatment-Resistant Schizophrenia

To date, clozapine is unique as it is the only evidence-based treatment for TRS with 60–70% of those treated showing a response and it appears superior to all antipsychotics, including other atypical antipsychotics, in treating this population (Chakos et al., 2001; Lally et al., 2016).

The pioneering CT study by Friedman et al. (1991) showed that the degree of prefrontal cortex reduction was inversely related to clozapine response. Subsequent CT and MRI studies replicated this finding, demonstrating that a lower level of prefrontal atrophy was associated with clozapine treatment response compared with clozapine non-responders (Honer et al., 1995; Konicki et al., 2001; Arango et al., 2003; Molina et al., 2003). However, others were unable to replicate these results (Bilder et al., 1994; Lauriello et al., 1998). Only the study performed by Molina et al. (2003) showed a correlation between psychotic symptoms improvement and temporal GM volume in TRS patients treated with clozapine, whereas disorganization symptoms improvement was inversely related to pretreatment hippocampal volume (Molina et al., 2003). Moreover, a longitudinal study (Chakos et al., 1995) showed that, over the course of 1-year, patients started on clozapine showed a reduction in caudate nucleus volume, whereas an increase was showed in those remaining treated with typical antipsychotics. These findings were replicated by two studies showing that clozapine use led to caudate nucleus volume reductions over 24 weeks (Scheepers et al., 2001a) and 52 weeks (Scheepers et al., 2001b).

Concerning functional neuroimaging, SPECT or PET studies showed a correlation between prefrontal and thalamus metabolic activity reductions and clozapine treatment, but it is uncertain whether these findings were related to clinical response (Molina Rodríguez et al., 1996; Molina et al., 2003, 2005). Nakajima et al. (2015) extensively reviewed these studies, showing no association between brain changes and clozapine response. Furthermore, in an MRS study, clozapine-responsive TRS patients showed that glutamate and glutamine concentrations were increased in the putamen and decreased in the dlPFC (Goldstein et al., 2015).

Several studies have investigated the relationship between genetic variants and response to clozapine, and several significant associations were reported with genes that are mainly involved in the dopaminergic, serotonergic and inflammation/immune systems. However, even with all of these relevant results, only three genetic variants, the Ser9Gly polymorphism of the *DRD3* gene previously cited in the dopaminergic system section, the functional non-synonymous coding region SNP 452His/Tyr (rs6314) of the *5-HT2A* gene, and the C825T variant of the G protein subunit beta 3 (*GNB3*) gene, have had significant findings independently replicated (Samanaite et al., 2018).

Moreover, a recent study has suggested the existence of a more severe, genetically based schizophrenia subgroup, for whom early intervention with clozapine can be considered. If confirmed from further research this finding may have important implications for clinical practice (Frank et al., 2015).

Despite all these demonstrations and the efficacy of clozapine in TRS, it is underprescribed in most countries (Lally et al., 2016). The explanations for this include worries of side effects, the inconvenience of therapeutic blood monitoring, and all potential fatal outcomes associated to clozapine use (Li et al., 2018). This means that the levels of use are far less than the about 50–60% of TRS patients who could benefit from it, although several studies highlight that clozapine remains the gold-standard treatment for TRS (Taylor, 2017).

## Conclusion and Future Perspectives

Although interesting data have come from pharmacogenetics, neuroimaging and the interaction of both fields of study, few converging findings are available that describe the antipsychotic treatment response and resistance mechanisms in schizophrenia (DeLisi and Fleischhacker, 2016). Based on the available evidence, the results from both neuroimaging and pharmacogenetic/pharmacogenomic studies point to an overlap in the neurobiological vulnerability risk factors influencing the antipsychotic drug response in schizophrenia and the risk factors underlying schizophrenia itself. Currently for TRS, not a single biological marker, both coming from neuroimaging or genetic studies, is available. Indeed, all researches carried out to date did not provide findings with strong requisites of reproducibility, specificity, robustness as well as clinical feasibility and cost-effectiveness. Consequently, it is difficult to delineate a model of pharmaco-resistance and a clear pathogenetic hypothesis. Several reasons are to address: (1) the definition of resistance for schizophrenia is still lack of a definitive consensus; (2) few studies are available on TRS and most are on clozapine. As in our review, we refereed mainly to non-response mechanisms that could be partly overlying the aetiopathogenesis of resistance; (3) most data come from *a priori* hypotheses studies focused on well-known pathways; (4) several methodological limitations in the existing literature, including lack of reliability data, clinical heterogeneity among studies, and inadequate study designs and statistics.

More investigations are necessary on this important topic, and future direction should be focused on GWAS on TRS that will permit to obtain results regarding the involvement of other pathways/systems rather than the usual to date investigated, allowing further targets of future neuroimaging studies.

We hope that technology development and the opportunity to carry out studies in clinically homogeneous patient samples could represent the opportunity to obtain predictive genetic testing for use in clinical practice. Moreover, drug repositioning associated to GWAS data and drug expression profiling (So et al., 2017), could be applied to severe psychiatric disorders as TRS. Regarding neuroimaging results to be clinically translatable, upcoming investigations require to be adequately powered and integrated with other biological markers. Further studies with large cohorts are needed for a better evaluation of the genetic contribution to the mechanisms underlying antipsychotic treatment response and resistance, hopefully in combination with non-biological markers, such as childhood trauma, which represent a clinically relevant factor for the development of TRS (Koga et al., 2017).

## Author Contributions

AV, AM, SB, GD, EG, PV, CT, and MG participated in the writing process of the first draft of the manuscript. AM and SB made literature search and independently reviewed electronic databases. AM, EG, and MG revised the pharmacogenetics and pharmacogenomic correlates in TRS, while AV, SB, GD, PV, and CT revised neuroimaging correlates in TRS. AV, AM, SB, and MG revised the final version of the manuscript. All authors contributed to reading and approving the final version of the manuscript.

## Conflict of Interest Statement

The authors declare that the research was conducted in the absence of any commercial or financial relationships that could be construed as a potential conflict of interest. The handling Editor declared a past co-authorship with one of the authors AV.

## Footnotes

1 http://www.ipap.org
